# Phytoremediation of particulate matter from indoor air by *Chlorophytum comosum* L. plants

**DOI:** 10.1007/s11869-014-0285-4

**Published:** 2014-08-09

**Authors:** H. Gawrońska, B. Bakera

**Affiliations:** 1 Laboratory of Basic Research in Horticulture, Faculty of Horticulture, Biotechnology and Landscape Architecture, Warsaw University of Life Sciences, 159 Nowoursynowska Str., 02-776 Warsaw, Poland; 2 Department of Plant Genetics Breeding and Biotechnology, Faculty of Horticulture, Biotechnology and Landscape Architecture, Warsaw University of Life Sciences, 159 Nowoursynowska Str., 02-776 Warsaw, Poland

**Keywords:** Indoor air, PM accumulation, PM categories, Size fraction, Spider plant, Waxes

## Abstract

Higher plants, including spider plants, are able to take up and degrade/detoxify various pollutants in the air. Although nearly 120 plant species have been tested for indoor air phytoremediation, to the best of the authors’ knowledge, data on particulate matter (PM) phytoremediation from indoor air are not yet available in literature. This work determined the ability of spider plants to take up PM, one of the most harmful pollutants to man, in the indoor air of five rooms housing different activities (a dental clinic, a perfume-bottling room, a suburban house, an apartment and an office). It was found that spider plants accumulate PM of both categories (water washable and trapped in waxes) and in all three size fractions determined and that the amount differed depending on the type of activity taking place in the particular rooms ranging from 13.62 to 19.79 μg/cm^2^. The amount of wax deposited on the leaves of plants grown in these rooms also differed (34.46–72.97 μg/cm^2^). The results of this study also demonstrated that the amount of PM accumulated on aluminium plates was always significantly lower than that accumulated on the plants’ leaves, showing that more than simply gravity forces are involved in PM accumulation on leaf blades.

## Introduction

People living in urban areas spend up to 85–90 % of their time indoors (Soreanu et al. 2013), often unaware that they might be continually exposed to air pollution. According to the United States Environmental Protection Agency (US EPA), indoor air pollution has been ranked among the top five risks to public health (Kobayashi et al. 2007; Wolverton 2008; Soreanu et al. 2013) as it has a negative impact on people’s health and property in urban areas all over the world (Kleeberger 2003). Sometimes, the level of air pollution indoors can be more than ten times higher than the outdoors, and in the case of some harmful substances, their concentrations can even exceed permissible norms by up to 100 times (Wolverton 2008). Brody (2001) reported that indoor concentrations of some cancerous chemicals are between 5 and even up to 70 times higher than outdoors, although the indoor concentration of pollutants is still lower than in industrial factories and heating power stations or next to busy roads (Wood 2003).

The quality of indoor air is an important issue because people inhale 6–10 l of air a minute, which amounts to 15,000 l/day (Wood et al. 2002), together with pollutants present in the air. Poor air quality interferes with learning and concentration in schools and can be a cause of health problems both to pupils and teachers (Daisey et al. 2003). All the pollutants mentioned contribute to runny noses, fever, coughs (Daisey et al. 2003), asthma (Daisey et al. 2003; Wolverton 2008), itchy skin, burning eyes (Wolkoff et al. 2006), headaches (Wood 2003; Tseng et al. 2005), nausea, dizziness (Costa et al. 1995; Wood et al. 2002; Tseng et al. 2005), cancers (Costa et al. 1995; Brody 2001; Wood et al. 2002; Tseng et al. 2005; Wolverton 2008) and even death (Brody 2001; D’Amato et al. 2002). Particulate matter (PM)_2.5_ alone is responsible for over 2 million deaths a year around the world (Silva et al. 2013). PM also causes damage to the circulatory and respiratory systems (Costa and Dreher 1997) and are also the second largest cause of lung cancer (European Environmental Agency (EEA) 2007) and autism (Volk et al. 2013).

In general, air pollutants found in the indoor air are categorised into volatile organic compounds (VOCs), inorganic gaseous compounds (ICs) and particulate matter (PM) (Soreanu et al. 2013). Major sources of indoor air pollution are shown in Table 1.

**Table 1:** Sources and type of pollution in the indoor

Sources of pollution	Types of pollution	Literature
Outdoor air	PM, nitrogen dioxide, carbon monoxide	D’Amato et al. 2002; Afshari et al. 2005
Buildings’ materials, glues, paints, furniture	Alcohols, benzene, formaldehyde and toluene	Kobayashi et al. 2007; Wolverton 2008
Home ware: carpets, wallpapers	PM, bacteria, fungi, mites, dust	Daisey et al. 2003; Wood 2003; Wolverton 2008
Electronic equipment: computers, televisions, monitors	Ammonia, benzene, toluene, trichloroethylene and PM	Afshari et al. 2005; Wolverton 2008
Activities of rooms’ users: frying meat, using fireplaces, ironing, cigarette smoking, cleaning	PM, nitrogen dioxide, carbon monoxide, benzene, formaldehyde	WHO 2000; Brody 2001; Afshari et al. 2005; Kobayashi et al. 2007; Wolverton 2008
Biological: man, animals, fungi, bacteria	Ammonia, acetone, alcohols, methane, nitrogen oxides, carbon monoxide and sulphur compounds, PM	Brody 2001; Afshari et al. 2005; Kobayashi et al. 2007; Wolverton 2008

PM has recently been considered to be one of the most dangerous health pollutants to human life (EEA 2007). PM is a mixture of solid and liquid phases and differs in size, origin and chemical compositions. PM found in indoor air, in addition to entering from outdoors, is also generated indoors by the use of domestic burners, heaters and fireplaces (WHO 2000; Brody 2001; D’Amato et al. 2002) and during frying (D’Amato et al. 2002; Afshari et al. 2005), ironing (Afshari et al. 2005), cleaning (Brody 2001; Wood et al. 2002), the presence and activity of members of the household (Brody 2001; Afshari et al. 2005; Kobayashi et al. 2007; Wolverton 2008) and pets (Daisey et al. 2003).

After emission, PM can be present in the air for a period ranging from a number of hours to several weeks and may stay in the emission place and also be transported in long distances (Farmer 2002). Based on its aerodynamic diameter, there are several classifications of PM. Most often in literature, the classification with four fraction sizes is cited: large—Ø 10–100 μm; coarse—Ø 2.5–10 um (PM_10_); fine—0.01–2.5 μm (PM_2,5_) and super (or ultra) fine—less than Ø 0.01 μm.

Heavy metals (Voutsa and Samara 2002), organic compounds such as polycyclic aromatic hydrocarbons (PAH) (Caricchia et al. 1999; Kaupp et al. 2000) and environmentally persistent free radicals (EPFRs) (Saravia et al. 2013) may settle on PM. According to Costa and Dreher (1997), the toxicity, harmfulness and negative impact of PM on health are due to the high content of heavy metals and their bioavailability and ease of accumulation in the cells of living organisms. Saravia et al. (2013) among others focused in particular on the harmful effects of EPFRs on infants.

Alongside conventional techniques to clean indoor air, environmental biotechnology such as phytoremediation, in which growing plants with associated microorganisms take up pollutants and degrade or detoxify them, may also be employed. Compared to the technical methods, phytoremediation technology is cheaper, environmentally friendly and can be used for a wider range of both organic and inorganic impurities. However, the time of purification is longer (Schwitzguébel 2000). In outdoor urban areas, the use of green canyons offers the possibility of lowering the level of PM by up to 60 %. However, the effectiveness of this depends on weather conditions such as wind speed (Pough et al. 2012). The efficiency of indoor phytoremediation depends on the chosen system, which can be passive (only planting in pots) or active (plants with filters, active carbon).

Studies have shown that ornamental plants have the ability to absorb, distribute and/or transport organic pollutants to microorganisms associated with higher plants living both in the rhizosphere (Wolverton and Wolverton 1996) and phyllosphere (Sorkhoh et al. 2011). Leaf size, structure, the ticker layer of waxes, pubescence and surface roughness usually correspond to a higher absorption of pollutants from both indoor and outdoor air (Peart 1992).

*Chlorophytum comosum* L. (spider plant) is among 120 plant species assayed for phytoremediation of pollutants from indoor air (Soreanu et al. 2013). It has the ability to remove formaldehyde, nitrogen dioxide, carbon oxide, ozone, benzene, toluene, cigarette smoke and ammonia (Peart 1992; Giese et al. 1994; Costa et al. 1995; Cornejo et al. 1999; Wolverton 2008). It has been shown by Giese et al. (1994) that the spider plant uses formaldehyde as a source of energy and carbon for biosynthesis of new molecules. The literature on particulate matter uptake from the air by outdoor-growing plants is very extensive, most probably because of this pollutant’s increasing negative impact worldwide on human health and the environment. However, there is an absence of data on the role of plants in PM phytoremediation from indoor air.

The objectives of this study were (i) to check whether the spider plant accumulates PM from indoor air and, if so, (ii) to establish whether the amount of accumulated PM and deposited waxes is affected by the activity taking place in particular rooms and (iii) to establish whether factors other than gravity play a role in PM accumulation on the plants’ leaves.

## Materials and methods

### Materials

Spider plants (*C. comosum* L.) planted in pots filled with horticultural substrate purchased from Tomaszewski Sp. z o.o. were used in this study. All plants selected for the experiment were at the same stage and free of insects and symptoms of phytopathogenic attack. In order to answer the question of whether factors other than gravity play a part in PM accumulation on the plants’ leaves, aluminium plates (AL-plates) were also used as a control surface for PM deposition.

### Study sites and sample collection

Plants growing in pots and the AL-plates were exposed for 2 months (October–December) to the indoor air of five rooms which differed in the activities taking place in them: (1) a dental clinic, (2) a perfume-bottling room, (3) a suburban house, (4) an apartment and (5) an office. There was no air conditioning in these rooms, so by carefully selecting where the plants were located, an attempt was made to ensure similar air conditions as far as possible in terms of light, temperature and humidity. Plants were placed on windowsills at a distance of ~20 cm from the pane of glass, and the windows were selected in a way that no trees and other buildings produced a shadow on the sills. Temperatures ranged between 18 and 23 °C depending on the time of day.

### Quantitative analysis of PM and epicuticular waxes

After 2 months of exposure, leaf samples were collected (about 350–400 cm^2^ for each room per replication), placed in properly labelled paper bags, transported to the laboratory and stored until analysis.

The mass of PM and wax was determined gravimetrically according to the methodology of Dzierżanowski et al. (2011). The PM was determined in two categories (first washed with water for surface PM, followed by washing with chloroform for in-wax PM). For the purposes of simplicity in this paper, surface PM and in-wax PM are denoted as sPM and wPM, respectively. Whatman (UK) filters Type 91, Type 42 and PTFE membrane were used to capture particles of <10, <2.5 and <0.2 μm, respectively. Filters before filtration were dried (PREMED KCW–100), stabilised for humidity and, after being passed through a deioniser gate (HAUG, Switzerland), were weighed using a microbalance with an accuracy of up to 0.00001 g (XS105DU, Mettler-Toledo International Inc., Switzerland). After filtration, drying, stabilising and passing through a deioniser gate (as above), the filters were weighed again. For the quantification of waxes, the chloroform during filtration was collected in pre-weighed beakers and evaporated. The masses of both PM and waxes were calculated as differences between the respective masses of the filters or beakers before and after filtration/evaporation. In order to express PM and waxes per square centimetre (which allows data from the examined rooms to be compared), the area of each leaf sample was determined using an image Analysis System (Skye Instruments Ltd, UK). PM was washed off from both surfaces of the leaves, but the amounts are expressed per unit leaf area for one surface only.

Since the surface of the AL-plates is very smooth and does not contain any fatty compounds or waxes, only water was used for washing to determine the amount of PM deposited on them. This means that only one category of PM was determined from the plates. PM from AL-plates was washed at the site of exposure (in the particular rooms).

### Statistical analysis

Data was subjected to one-way ANOVA (analysis of variance) using StatGraphics Plus 4.1 software (StatPoint Technologies, Inc., USA). The significance of differences between mean values for given rooms was estimated using Tukey’s Honestly Significant Difference (HSD) test. Data presented is mean ± SE, *n* = 5.

The experiment was repeated twice, with two replications in experiment 1 and five replications in experiment 2. The additional combination (AL-plates) was also included in the second experiment. The nature of the differences between the rooms, the PM categories and size fractions in both experiments was very similar. To present the results in this article, it was decided that the data from experiment 2 was more reliable and informative (more replications and combinations) and would therefore be used.

## Results

### Amount of PM and waxes accumulated on the leaf blades of the spider plant

**Fig. 1:**
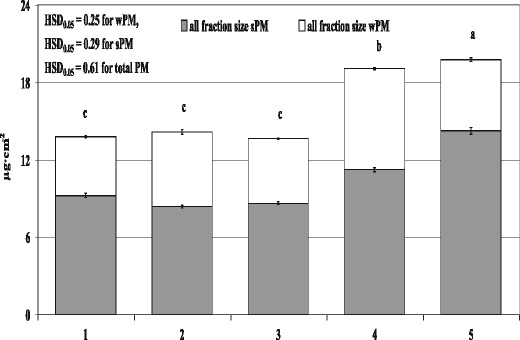
Amount of total PM (sPM + wPM) accumulated on leaf blades of spider plants (*Chlorophytum comosum* L.) growing for 2 months in five rooms differing in activities (*1* dental clinic, *2* perfume-bottling room, *3* suburban house, *4* apartment, *5* office). Data is mean ± SE, *n* = 5. *Bars marked with different letters* represent significant differences (*p* < 0.05) in total PM

The total amount of PM accumulated on the leaves of the spider plant differed between the rooms examined and ranged from 13.62 to 19.79 μg/cm^2^, with the largest and smallest amounts recorded for the office and the suburban house, respectively (Fig. 1). The amount of total PM accumulated on leaves grown in the office and the apartment was significantly higher than in the other three rooms. In each of the rooms, more PM was recorded for the category of sPM than wPM (Fig. 1), whose contributions to total PM (average for all rooms) were 64.24 and 35.76 %, respectively.

**Fig. 2:**
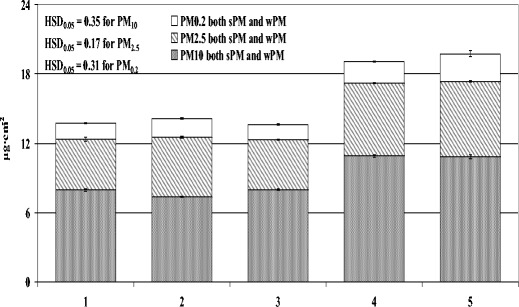
Amount of total PM (sPM + wPM), taking into account the size fractions, accumulated on leaf blades of spider plants (*Chlorophytum comosum* L.) growing for 2 months in five rooms differing in activities (*1* dental clinic, *2* perfume-bottling room, *3* suburban house, *4* apartment, *5* office). Data is mean ± SE, *n* = 5. *Bars marked with different letters* represent significant differences (*p* < 0.05) in total PM

The quantity of PM of particular size fractions also differed. In every room, the greatest proportion to total PM was large PM (10–100 μm) and the smallest was fine PM (0.2–2.5 μm) at 56.04 and 10.74 %, respectively (Fig. 2). This was also true when looking at the size fractions in the category of sPM, but not for wPM. The amount of fine PM, with few exceptions, was higher in the category of wPM when compared to sPM (58.13 vs. 41.87 %, data not shown).

**Fig. 3:**
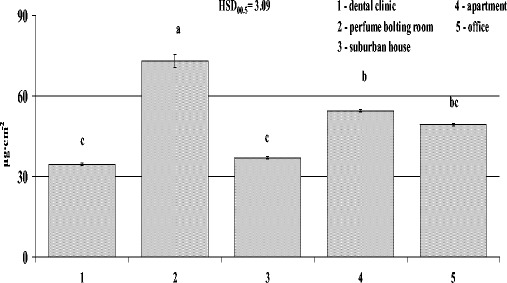
Amount of epicuticular waxes deposited on the surface of leaf blades of spider plants (*Chlorophytum comosum* L.) growing for 2 months in five rooms differing in activities (*1* dental clinic, *2* perfume-bottling room, *3* suburban house, *4* apartment, *5* office). Data is mean ± SE, *n* = 5. *Bars marked with different letters* represent significant differences (*p* < 0.05)

The quantity of epicuticular waxes deposited on leaves, similarly to PM, was different in each room. The greatest amount of waxes deposited on leaves (more than double the smallest amount) was recorded for plants grown in the perfume-bottling room, while the amount of wax on leaves of plants growing in the dental clinic was the lowest (72.97 vs. 34.46 μg/cm^2^, respectively) (Fig. 3).

### Amount of PM deposited on aluminium foil plates

**Fig. 4:**
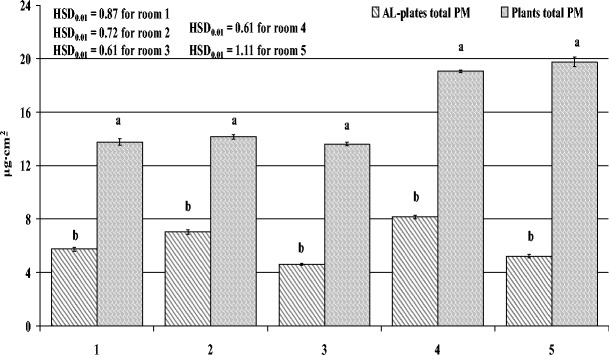
Amount of total PM accumulated on leaf blades of spider plants (*Chlorophytum comosum* L.) and deposited on aluminium plates during 2 months of exposure to indoor air in five rooms differing in activities (*1* dental clinic, *2* perfume-bottling room, *3* suburban house, *4* apartment, *5* office). Data is mean ± SE, *n* = 5. ANOVA was conducted for data from every room separately. *Bars marked with different letters* represent significant differences (*p* < 0.01)

**Fig. 5:**
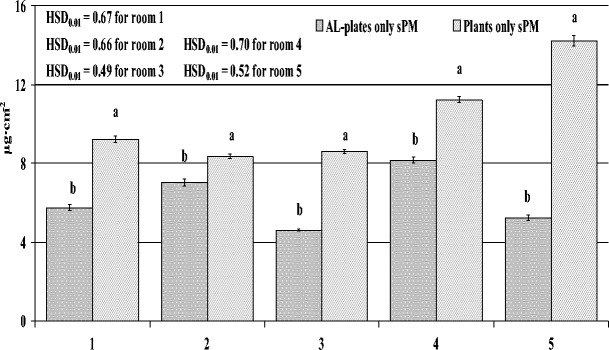
Amount of total surface PM, taking into account the size fractions, accumulated on leaf blades of spider plants (*Chlorophytum comosum* L.) and deposited on aluminium plates during 2 months of exposure to indoor air in five rooms differing in activities (*1* dental clinic, *2* perfume-bottling room, *3* suburban house, *4* apartment, *5* office). Data is mean ± SE, *n* = 5. ANOVA was conducted for data from every room separately. *Bars marked with different letters* represent significant differences (*p* < 0.01)

The amount of PM deposited on the AL-plates, similar to the PM accumulation on the leaf blades of the spider plants, differed between the rooms surveyed, with the highest being in the apartment and the lowest in the suburban house at 8.15 and 4.60 μg/cm^2^, respectively. The amount of total PM accumulated on leaves was significantly higher when compared with the amount deposited on the AL-plates (Fig. 4). Moreover, the amount of PM deposited on the AL-plates was also significantly lower when compared with the amount of sPM (washed off leaves just with water) (Fig. 5). As with PM on leaf blades, on AL-plates in every room, the greatest amount was large PM (68.34 %), while fine PM always accumulated in the smallest amounts (7.33 %) (data not shown).

## Discussion

As has already been stated, PM is one of the most harmful air pollutants to human health and is present both outdoors and indoors. In numerous studies, it has been shown that vegetation that accumulates PM improves outdoor air quality and reduces health risks (Pough et al. 2012; Soreanu et al. 2013). The results from this study clearly showed that spider plants (*Chlorophytum comosum* L.), well known for absorbing and detoxifying or degrading many harmful compounds such as NO_x_, CO, formaldehyde, benzene and others (Wolverton and Wolverton 1993; Giese et al. 1994; Costa et al. 1995; Cornejo et al. 1999; Wolverton 2008), are also able to accumulate PM.

PM of both categories, i.e. sPM and wPM, as well as all three determined size fractions were found on leaves of this species. The quantity of total sPM and wPM accumulated on leaf blades depended on the kind of activity taking place in the surveyed rooms and their locations. The lowest amount of PM accumulated on leaves was, as expected, recorded in plants growing in the house in Zielonka. This is due to fact that Zielonka is a suburb of Warsaw with no industrial activity and only low levels of traffic on the nearby roads. The highest amount of PM accumulated on the leaves of plants growing in the office and the apartment can be explained by (i) the location of these rooms (both are in buildings in Warsaw next to roads with heavy traffic) and (ii) the type of activities (printers and photocopies were heavily used in the office, and in the apartment, residents undertook high levels of physical activity and used a stationary bike on a daily basis).

In this study, the share of sPM in the total amount of PM was, in most cases, greater than that of wPM. These results are in agreement with the results of previous studies carried out in the authors’ laboratory on 52 woody plant species growing outdoors (Dzierżanowski et al. 2011; Popek et al. 2011, 2013, 2014), but not with data reported for small-leaved limes grown in Warsaw (Nawrot et al. 2011) or for some species grown in Norway (Sæbø et al. 2012). sPM was also in greater proportion than wPM in the case of large PM and coarse PM, but not usually in the case of fine PM.

The results of this work also showed that irrespective of the activity taking place in the particular rooms, of the three size fractions, the amount of large PM was greatest while the amount of fine PM was the smallest, with the exception of wPM. These results are in line with data on 51 species of trees and shrubs grown outdoors in urban areas (Dzierżanowski et al. 2011; Popek et al. 2011; Sæbø et al. 2012; Popek et al. 2013, 2014).

Fine PM was found at higher levels in the category of wPM than sPM and as such was more firmly attached to the leaves. This means that fine PM is phytostabilised to a greater extent than coarse and large PM. It is worth underlining that phytostabilisation of fine PM in wax lowers the risk to human health, because fine PM and ultra fine PM (which were not determined separately in this study) are most harmful to people. This is because they easily reach the alveolar region of the respiratory tract and can penetrate into the bloodstream and be transported in the blood to all organs (Nemmar et al. 2002). Moreover, highly toxic heavy metals are carried on fine PM (Voutsa and Samara 2002) along with PAH (Caricchia et al. 1999; Kaupp et al. 2000) and EPRFs (Saravia et al. 2013), making them even more toxic.

In this study, it was also shown that the amount of waxes deposited on leaf blades differed depending on the room in which the plants were growing. The highest amount was in the perfume-bottling room. This is probably because this activity is associated with a higher level of VOCs in the air in comparison with the other rooms, which can in turn generate stress for the plants. Popek et al. (2014) reported higher amounts of wax deposited on the leaves of trees growing at shorter distances from the source of PM emission as compared to those growing at further distances, for example inside the parks/forest.

Although the amount of wax deposited on leaves differed significantly between plants growing in various rooms, similar to the data of Dzierżanowski et al. (2011), Sæbø et al. (2012) and Popek et al. (2013), no correlation was found between the amount of wax and the quantity of accumulated PM.

In every room examined, the amount of PM accumulated on the leaves of the spider plants was always significantly higher than the amount of PM deposited on the AL-plates. This was true irrespective of whether comparisons were being made, in the case of leaf blades, between total PM and AL-plates or just sPM (i.e. the part washed with water) and AL-plates. It clearly demonstrates that gravitation is not the sole factor involved in PM accumulation on the plants’ leaves. This is in line with the statement by Pough et al. (2012) that the dry deposition of air pollutants among other things depends on the nature of the surface and is generally higher with vegetation than with other surfaces because of the metabolic uptake by plants, the stickiness of the leaf surface, the large surface area of plants (when compared to the ground area occupied by plants, LAI) and their aerodynamic properties. There is also data showing that the efficiency of pollutant uptake by plants differs depending on genotype, roughness of leaves, pubescence, the amount of wax, the topography of leaves on stems creating turbulence and the distance from the source of emission (Popek et al. 2013 and articles discussed in it).

In the case of spider plants growing indoors, it is highly probable that wax deposited on leaf blades plays an important role. Perhaps there are interactions between the waxes of spider plant leaves and the hydrophobic nature of PAH (which settles on PM). On the other hand, since the amount of PM did not correlate with the amount of wax, other forces must also be involved in PM accumulation on leaves. It is possible that the metabolic uptake underlined by Pough et al. (2012) plays an important role, but electrostatic forces between heavy metal ions settling on PM and leaf blades cannot be ruled out. To answer this question, more detailed and specifically designed studies are needed.

While at this stage there is no answer to questions about the forces involved in PM accumulation on the leaves of plants, this was not actually the objective of this study. The intention was to establish whether indoor plants also accumulate PM and, to the authors’ best knowledge, this is the first data to be presented in literature on PM phytoremediation from indoor air.

This study has proved that spider plants growing indoors accumulate PM. Although growing plants in pots is, in addition to the other positive functions of a plant, only a passive way of cleaning air, even so it contributes to improving air quality. In order to make air phytoremediation more effective, combinations of plants with associating microorganisms (Schwitzguébel 2000) or botanical filtration with biotrickling filters (Soreanu et al. 2013) can be employed besides conventional, albeit more expensive technologies. Therefore, indoor plant cultivation deserves to be popularised more widely since it reduces the risk to human health.

### Summary and conclusions

- Spider plants (*Chlorophytum comosum* L.) grown indoors accumulate particulate matter of both categories and all size fractions, irrespective of their location and the type of activity taking place in the examined room. They therefore phytoremediate PM from indoor air.
- The amount of PM accumulated on leaves depends on the kind of activity taking place in the particular room.
- Fine PM, the most harmful to human health, is accumulated to a greater extent as wPM than sPM because it is attached more tightly to leaves and is thereby phytostabilised more effectively. This reduces the risk to human health to a greater extent.
- Of the three size fractions examined, large PM constitutes the greatest proportion of PM accumulated on plants’ leaves.
- Accumulation of particulate matter on leaves involves factors/forces other than gravitation.
